# ABCB5⁺ mesenchymal stem cells enable high-ratio cellular augmentation of bone grafts without loss of osteogenic function

**DOI:** 10.1007/s00068-026-03311-4

**Published:** 2026-09-03

**Authors:** Jakob Hofmann, Tim Niklas Bewersdorf, Ulrike Sommer, Kai Borcherding, Karsten Thiel, Christoph Ganss, Matthias Gerstner, Markus H. Frank, Gerhard Schmidmaier, Mark Andreas Kluth, Tobias Grossner

**Affiliations:** 1 Clinic for Trauma and Reconstructive Surgery, Center for Orthopedics, Trauma Surgery and Paraplegiology, University Hospital Heidelberg, Schlierbacher Landstrasse 200a,, 69118 Heidelberg, Germany; 2 Faculty of Medicine, Heidelberg University, 69120 Heidelberg, Germany; 3 Fraunhofer Institute for Manufacturing Technology and Advanced Materials IFAM, 28359 Bremen, Germany; 4 RHEACELL GmbH & Co. KG, 69120 Heidelberg, Germany; 5 Transplant Research Program, Boston Children’s Hospital, 300 Longwood Avenue, Boston, MA 02115 USA; 6 Harvard Stem Cell Institute, 7 Divinity Avenue, Cambridge, MA 02138 USA; 7 Department of Dermatology, Brigham and Women’s Hospital, 75 Francis Street, Boston, MA 02115 USA; 8 School of Medical and Health Sciences, Edith Cowan University, 270 Joondalup Drive, Joondalup, WA 6027 Australia

## Abstract

Autologous bone grafting remains the standard for reconstruction of bone defects but is constrained by limited graft volume and loss of osteogenic capacity when expanded with acellular materials. We hypothesized that allogeneic ABCB5⁺ mesenchymal stem cells (MSCs) can serve as a biologic cell source to augment bone marrow-derived MSC (BM-MSC) populations without impairing osteogenesis. Primary human BM-MSCs from five donors were combined with GMP-manufactured ABCB5⁺ MSCs at graded replacement ratios (0–95%) and cultured under osteogenic conditions for 21 days. Osteogenic potential was evaluated using complementary assays of mineralization, biochemical activity, and matrix composition. Robust osteogenesis was maintained across all groups, with cultures containing 50% ABCB5⁺ MSCs demonstrating comparable functional osteogenic activity to BM-MSC controls, including similar ^99m^Tc-HDP uptake (2.96 vs. 2.68 MBq) and calcium–phosphate ratios (1.2 vs. 1.1). Higher substitution levels showed a progressive decline in osteogenic output, consistent with dilution of BM-MSC-driven effects. These findings establish a quantitative threshold for biologic augmentation and demonstrate that substantial replacement of BM-MSCs is feasible without loss of function. This approach may enable cell-based expansion of bone grafts and supports further evaluation in translational in vivo models.

## Introduction

A relevant percentage of long-bone fractures (up to 10%) are under the risk of becoming a non-union, which, per definition, cannot resolve without further intervention [1]. Whenever possible, the first-line surgical therapy should aim for a biologic reconstruction of the non-union and the accompanied bone defect to ensure a long-lasting positive outcome for the patient. The current clinical standard is still the transfer of an autologous bone graft (ABG) into the defect. To ensure an optimal integration of the ABG into the bone, the induced-membrane technique (IMT) as described by Masquelet is regularly being applied. Here, the ABG is introduced into the bone during the second step of the procedure, after a biologically active and vascularized membrane has been formed [2]. The following ABG-based bone-healing is a complex process with numerous relevant molecular and biomechanical mechanisms interlinking. These mechanisms are condensed into a clinically applicable summary by the diamond concept [3]. One of the main pillars of the diamond concept is the presence of a sufficient concentration of osteogenically active cells.

Under normal circumstances, an adequate number of osteogenic cells is naturally contained within an ABG, which is commonly taken from the iliac crest or the medullary canal of long-bones, harvested with the reaming-irrigation-aspiration (RIA) procedure [4, 5]. While this form of treatment has been successfully used for decades, it also comes with certain limitations, chief among which is the limited reservoir of autologous bone material [6, 7]. Especially for patients with large defects or requiring multiple revision surgeries and re-grafting, the availability of biologic material will in all likeliness be exhausted. Therefore, a complete filling of the defect with the ABG, which is always the clinical aim to ensure an optimal outcome, would not be possible in these situations, setting a limit to the reconstruction potential [8].

To overcome the issues of a limited cell reservoir, clinicians are commonly using acellular materials such as decellularized bone grafts or synthetic bone grafts like calcium sulfate and tricalcium phosphate. These are applied as expanders together with the ABGs to augment the overall volume, so the defect can be fully filled [9]. However, this augmentation with acellular materials entails a number of drawbacks, notably the dilution of cells with biologically inactive material which results in a reduction of the overall osteogenic potential which can result in a complete failure of the surgical procedure [9]. Usually, clinicians aim not to exceed a graft augmentation of more than 30% with synthetic materials to retain its biologic capabilities [2].

Hence, whenever a synthetic augmentation is performed, a biologic augmentation should be considered simultaneously, with the goal of retaining the optimal regenerative potential of the graft. As autologous blood-derived products (ABPs) like platelet-rich plasma (PRP) and platelet-rich growth factors (PRGF) usually do not contain cells capable of bone healing, bone-marrow aspirate concentrate (BMAC) is currently the only available solution for an adequate biologic augmentation. As BMAC contains mesenchymal stem cells, growth factors and anti-inflammatory cytokines, it addresses three of the five pillars of the diamond concept. However, this method is associated with procedural risks for the patient, limited availability of autologous BM-MSCs and heterogeneous clinical results. This heterogeneity is largely attributable to the lack of standardized harvesting and processing procedures, making the biologic potency of the cell product and therefore the clinical success difficult to predict [10–12].

In essence, there is a high unmet medical need for an allogenic cell source for bone graft augmentation, which is clinically safe, capable of supporting the pillars of the diamond concept and is readily available in unlimited quantities without any additional harvest-associated risks for the patient.

ABCB5⁺ mesenchymal stem cells (ABCB5^+^ MSCs) are a population of human dermal stem cells that express the ATP-binding cassette member B5 (ABCB5). They meet the minimal criteria established by the International Society for Cellular Therapy (ISCT) for defining mesenchymal stem cells, including the ability to differentiate into osteogenic, chondrogenic, and adipogenic lineages (“trilinear differentiation”) [13–15]. Furthermore, ABCB5⁺ MSCs have been shown to secrete pro-angiogenic factors, including vascular endothelial growth factor (VEGF), and to exert immunomodulatory effects through modulation of the local inflammatory response. These biological properties are considered relevant to tissue regeneration and repair [16].

ABCB5⁺ MSCs are manufactured as an allogeneic advanced therapy medicinal product (ATMP) under Good Manufacturing Practice (GMP) conditions and are intended as an off-the-shelf cell therapy product [17]. Clinical studies have investigated their systemic and topical use in inflammatory diseases such as epidermolysis bullosa and chronic venous ulcers [18, 19]. Over all clinical studies and preclinical safety studies, ABCB5^+^MSCs have demonstrated a favorable safety profile, with no evidence of treatment-related tumorigenicity or toxicity after systemic or topical administration [18–20]. While the clinical safety and efficacy of ABCB5⁺ MSCs have been investigated across several inflammatory indications, their potential for the treatment of bone defects and the promotion of bone regeneration has not yet been evaluated in preclinical or clinical studies.

Taking their biologic properties into account, ABCB5^+^ MSCs may represent a suitable allogeneic source to augment BM-MSCs in bone repair. Their osteogenic, pro-angiogenic and anti-inflammatory properties suggest that they may support the osteogenic potential of BM-MSCs, making them a promising cell source for biologic augmentation of ABGs.

The aim of the present study was to investigate whether ABCB5^+^ MSCs can augment human BM-MSCs during osteogenic differentiation. Therefore, a structured assessment of the functional osteogenic potential of different BM-MSC/ABCB5^+^ MSC ratios was evaluated using osteogenic in vitro assays to provide a basis for possible future in vivo applications of ABCB5^+^ MSCs for bone healing.

## Materials and methods

### Study at a glance

The study was approved by the Ethic committee of the Medical Faculty, Heidelberg University, Germany (Protocol number S-363/2020). The study was conducted in accordance with the ethical principles for medical research involving human participants as outlined in the Declaration of Helsinki (revised at the 75th WMA General Assembly, Helsinki, Finland, October 2024). All patients provided informed written consent. A consent for publication is not applicable as our manuscript does not include any identifying images or other personal or clinical details of participants.

**Fig. 1:**
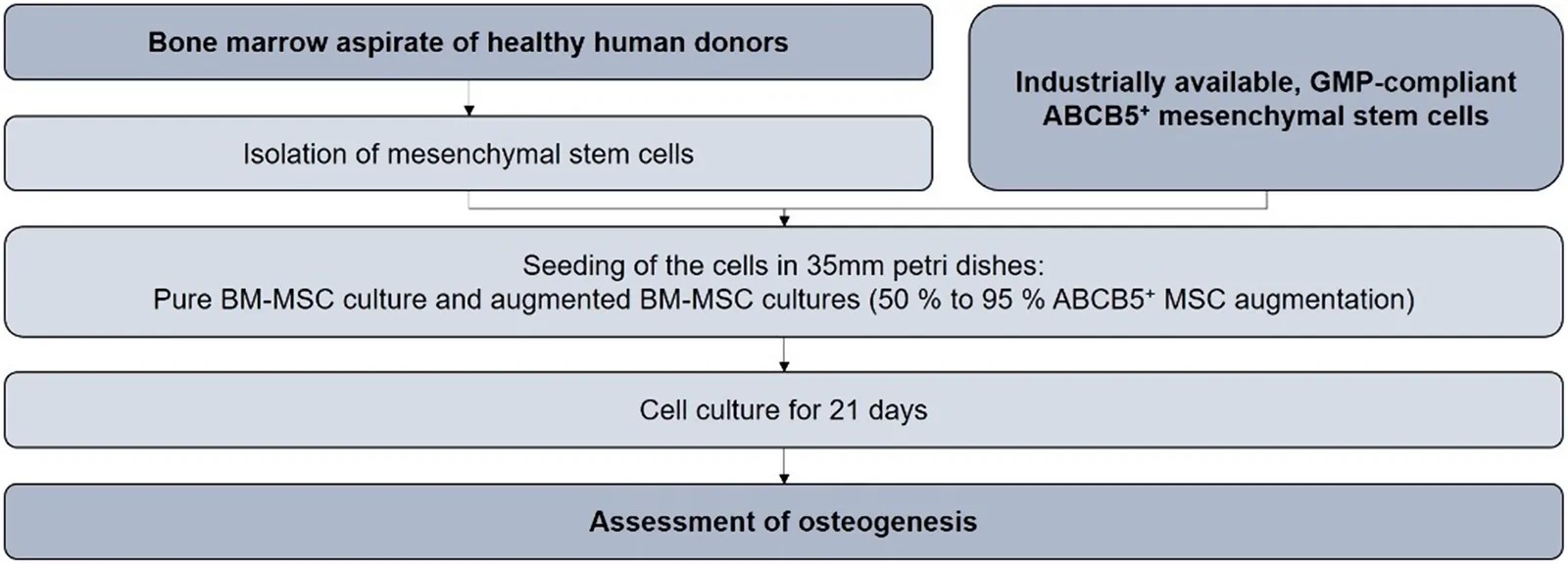
Overview over the study design

BM-MSCs were sourced from the proximal femur cavity of human donors and not pooled. Pooled GMP-grade ABCB5^+^ MSCs were provided by the manufacturer. Baseline functional osteogenic potential was defined by an osteogenically induced cell culture containing 100% BM-MSCs (see Fig. 1). Subsequently, cellular augmentation with ABCB5^+^ MSCs was carried out, starting with replacing 50% BM-MSCs with ABCB5^+^ MSCs and then increasing the cellular ABCB5^+^ MSC fraction in 5% increments until a 95% ABCB5^+^ MSC proportion was reached, resulting in 11 different groups in total (see Table 3). Each group was chemically differentiated towards the osteogenic lineage for three weeks while a corresponding non-osteogenic negative control was cultured in parallel.

To determine the functional osteogenic potential of the augmented individual cell cultures, the parameters in Table 1 were assessed for each group and then statistically compared to the baseline data of group 1, containing 100% BM-MSCs:

**Table 1:** Parameters measured to determine osteogenic potential

Target osteogenic marker	Relevance	Principle of Detection	Method of Measurement
Alkaline phosphatase	Early osteogenic differentiation marker	Detection in cell culture medium supernatant	Photometric measurement (Siemens Atellica Solution, Siemens, Erlangen, Germany)
Osteocalcin	Organic extra-cellular matrix formation marker	Detection in cell culture medium supernatant	Immunoluminescence Assay (Roche cobas pro e801, Roche, Penzberg, Germany)
Soluble calcium and phosphate	Extracellular matrix mineralization marker	Detection in cell culture medium supernatant	Photometric measurement (Siemens Atellica Solution, Siemens, Erlangen, Germany)
Insoluble calcium phosphate mineral	Extracellular matrix mineralization marker	Measurement of inorganic mineral via complexion of calcium	Alizarin Red staining
		Measurement of inorganic mineral via binding to phosphate	Radioactive ^99m^Tc-HDP labelling
		Proof of formation of hydroxyapatite via measurement of Ca/P ratio	Energy dispersive X-ray spectroscopy (EDX) (Oxford Instruments, Abingdon, UK)

### Sourcing of BM-MSCs

Bone marrow aspirate of *n* = 5 healthy adult human donors was harvested from the proximal femoral cavity during elective hip arthroplasty under general anesthesia after written informed consent was given. The aspirate was collected in a 20 mL syringe (BD, Heidelberg, Germany) containing 1,000 IU heparin (B. Braun, Melsungen, Germany). After the samples were diluted and washed with PBS (Gibco, Frankfurt, Germany), the mononuclear fraction was isolated by performing a Ficoll gradient (Ficoll-Paque-PLUS, Cytiva, Freiburg, Germany). The cells were then seeded in a T-175 polystyrene cell culture flask (Falcon, Kaiserslautern, Germany) with a density of 500,000 cells/cm² and cultured in a humidified CO_2_ atmosphere at 37 °C in expansion medium (DMEM-HG (Gibco) with 10% FCS (Sigma, Schnelldorf, Germany) and 1% penicillin/streptomycin (Sigma)). After 48 h, all flasks were washed with PBS to remove non-adherent cells. The remaining cells were defined as generation P_0_ human mesenchymal stem cells according to the minimal criteria of the ISCT [15, 21]. When 90% confluence was reached, cells were split into two new flasks. This process was repeated until a sufficient number of cells was available for the experiment. To reveal any influence derived from inter-donor osteogenic variability, thus mimicking future in vivo applications as closely as possible, the BM-MSCs were not pooled. The age and gender of the respective donors can be found in the following table (see Table 2):

**Table 2:** Donor characteristics

Donor	Age at time of harvest	Gender
Donor 1	75 years	Female
Donor 2	71 years	Male
Donor 3	64 years	Female
Donor 4	58 years	Female
Donor 5	86 years	Male

### Sourcing of ABCB5^+^ MSCs

ABCB5^+^ MSCs were sourced directly from the manufacturer (RHEACELL GmbH & Co. KG, Heidelberg, Germany) and provided in sterile frozen aliquots (P_11_). Prior to shipment, the cell product fulfilled predefined GMP specifications for batch release, including a potency assay regarding their anti-inflammatory capabilities, a VEGF secretion assay under hypoxic culture conditions and an in vitro tube formation assay to estimate the blood vessel-forming capacity of each cell batch produced [22].

### Osteogenic differentiation

11 groups were defined for this experiment using non-pooled-MSCs from five healthy human donors (*n* = 5) and pooled ABCB5^+^ MSCs.

**Table 3:** Experimental groups

Group (*n* = 5) & Condition	Percentage BM-MSCs	Percentage ABCB5^+^ MSCs
Group 1 - Osteogenic (OM)	100%	-
Group 1 - Control (CTRL)	100%	-
Group 2 - Osteogenic (OM)	50%	50%
Group 2 - Control (CTRL)	50%	50%
Group 3 - Osteogenic (OM)	45%	55%
Group 3 - Control (CTRL)	45%	55%
Group 4 - Osteogenic (OM)	40%	60%
Group 4 - Control (CTRL)	40%	60%
Group 5 - Osteogenic (OM)	35%	65%
Group 5 - Control (CTRL)	35%	65%
Group 6 - Osteogenic (OM)	30%	70%
Group 6 - Control (CTRL)	30%	70%
Group 7 - Osteogenic (OM)	25%	75%
Group 7 - Control (CTRL)	25%	75%
Group 8 - Osteogenic (OM)	20%	80%
Group 8 - Control (CTRL)	20%	80%
Group 9 - Osteogenic (OM)	15%	85%
Group 9 - Control (CTRL)	15%	85%
Group 10 - Osteogenic (OM)	10%	90%
Group 10 - Control (CTRL)	10%	90%
Group 11 - Osteogenic (OM)	5%	95%
Group 11 - Control (CTRL)	5%	95%

All cultures were seeded at 8,300 cells/cm² (80.000 cells per dish) in 35 mm petri dishes (Corning). To create the augmented cultures for groups 2 to 11 as shown in Table 3, ABCB5^+^ MSCs (generation P_11_, RHEACELL) were blended with BM-MSCs in a 5 mL Eppendorf tube (Eppendorf, Hamburg, Germany) at different proportions before seeding. For each group, two duplicates were created: the osteogenic condition (OM) received osteogenic medium (DMEM-LG with 10% FCS (Sigma), 1% penicillin/streptomycin (Sigma) and containing the osteogenic supplements 100 nmol/L dexamethasone (Sigma), 50 µmol/L L-ascorbic acid (Sigma) and 10 nmol/L β-glycerophosphate (Sigma)), while the control condition (CTRL) received control medium (DMEM-LG with 10% FCS (Sigma) and 1% penicillin/streptomycin (Sigma)). After 3 weeks, the culture medium supernatant was frozen at -20 °C for later analysis (1 mL per petri dish) before the cultures were terminated.

### ^99m^Tc-HDP labelling

The amount of calcium phosphate mineral formed at the bottom of the cell culture dish was assessed after 3 weeks by labelling it with the diphosphonate-bound radioactive tracer ^99m^Tc-HDP. In vitro, ^99m^Tc-HDP binds highly sensitively to phosphate complexes, thus indicating the presence of hydroxyapatite [23–25]. The method was previously validated by correlation analysis with quantitative Alizarin Red staining, the gold standard for the histological assessment of hydroxyapatite and can be seen as at least equally sensitive [26]. For the labelling process, ^99m^Tc-HDP was solved in 0,9% NaCl (B. Braun) to get to a concentration of 5.0 MBq per mL. Prior to the application of the tracer, the cell culture media was removed and the dish air dried. Each dish was then incubated with 1 mL of tracer aliquot at room temperature for 15 min. Then, the tracer was removed and the dishes were washed twice with PBS (Gibco). To quantify the remaining bound activity, the dishes were placed in the detector chamber of a dose calibrator (Activimeter ISOMED 1010, Nuklear-Medizintechnik Dresden GmbH, Dresden, Germany) and the detection window was set to γ-decay/^99m^Tc-HDP (see Table 1).

### Analysis of cell culture medium

The frozen culture medium samples obtained from the supernatant of the petri dishes after 3 weeks of cell culture were thawed and chemically analyzed by the central laboratory of Heidelberg University Hospital (see Table 1) using validated measuring methods. Alkaline phosphatase activity as well as calcium and phosphate concentration were assessed using Photometric measurement (Siemens Atellica Solution, Siemens, Erlangen, Germany) and osteocalcin concentration was assessed using an Immunoluminescence Assay (Roche cobas pro e801, Roche, Penzberg, Germany).

### Alizarin Red staining

0.5 g of Alizarin Red (Waldeck, Münster, Germany) were solved in 100 mL of distilled water. The solution was than adjusted to a pH of 4.1 using ammonia hydroxide. Representative dishes (*n* = 1) of both the osteogenic and control condition of groups 1 (100% BM-MSCs/0% ABCB5^+^ MSCs), 2 (50% BM-MSCs/50% ABCB5^+^ MSCs), 6 (30% BM-MSCs/70% ABCB5^+^ MSCs) and 10 (10% BM-MSCs/90% ABCB5^+^ MSCs) were then incubated for 10 min at room temperature with this solution before being washed 5 times with PBS (Gibco) to remove non-bound staining solution. The dishes were than qualitatively assessed by a camera to assess macroscopic distribution of the stain as well as under a light microscope. In a second step, to quantify the staining, all dishes were incubated with 0.5 mL of 10% CPC (Karl Roth, Karlsruhe, Germany) at room temperature. After that, 20 µL of the solution were either transferred directly into a 96-well-plate (control condition) or diluted 1:10 with CPC before being transferred (osteogenic condition). Air bubbles were carefully removed with the tip of a pipette. Measurement of the extinction was performed at 405 nm using a photometer with plate-reader (Thermo Fisher Scientific, Eindhoven, Netherlands).

### Energy dispersive X-ray spectroscopy (EDX)

To assess the composition of the calcium phosphate complexes formed, an EDX-analysis was carried out on representative dishes (*n* = 1) of the osteogenic condition of groups 1 (100% BM-MSCs/0% ABCB5^+^ MSCs), 2 (50% BM-MSCs/50% ABCB5^+^ MSCs) and 10 (10% BM-MSCs/90% ABCB5^+^ MSCs). Elemental analysis with a focus on phosphate and calcium was investigated with an Oxford X-Max80 silicon-drift detector (Oxford instruments, Abingdon, UK) attached to a scanning electron microscope with an energy resolution of 124 eV at Mn-Kα.

### Statistics

Because of the relatively small sample size with *n* = 5 and 11 groups, no Levene test was used to determine variance homogeneity as this test tends to be too conservative for small sample sizes [27]. Instead, we calculated statistical differences between the groups using a Welch-ANOVA and, if required, Games-Howell post-hoc testing. Because this ANOVA analyses is generally considered robust against violation of normal distribution, we did not conduct testing for normal distribution [28]. The global significance level was set to α = 0.05. For the sub-analyses of the osteogenic and control conditions, respectively, the global significance level was divided by the number of conditions, leading to a significance level of α = 0.025 for these analyses. To identify the influence of age and gender on the osteogenic potential as measured by the non-destructive assessments, a linear mixed model was performed. In the instances, where this model identified a significant influence, a linear correlation was also computed to quantify the extent of the influence. Statistical analysis was performed computer-aided (SPSS Statistics Version 29, IBM, Armonk, New York, USA).

## Results

*To improve both clarity and readability*,* the cells’ proportions are indicated in brackets behind each group mentioned in a shortened form. E.g.*,* “group 10 OM (10% BM-MSCs/90% ABCB5*^*+*^ *MSCs)” is abbreviated to “group 10 OM (B.10/A.90)”.*

### ^99m^Tc-HDP labelling

**Fig. 2:**
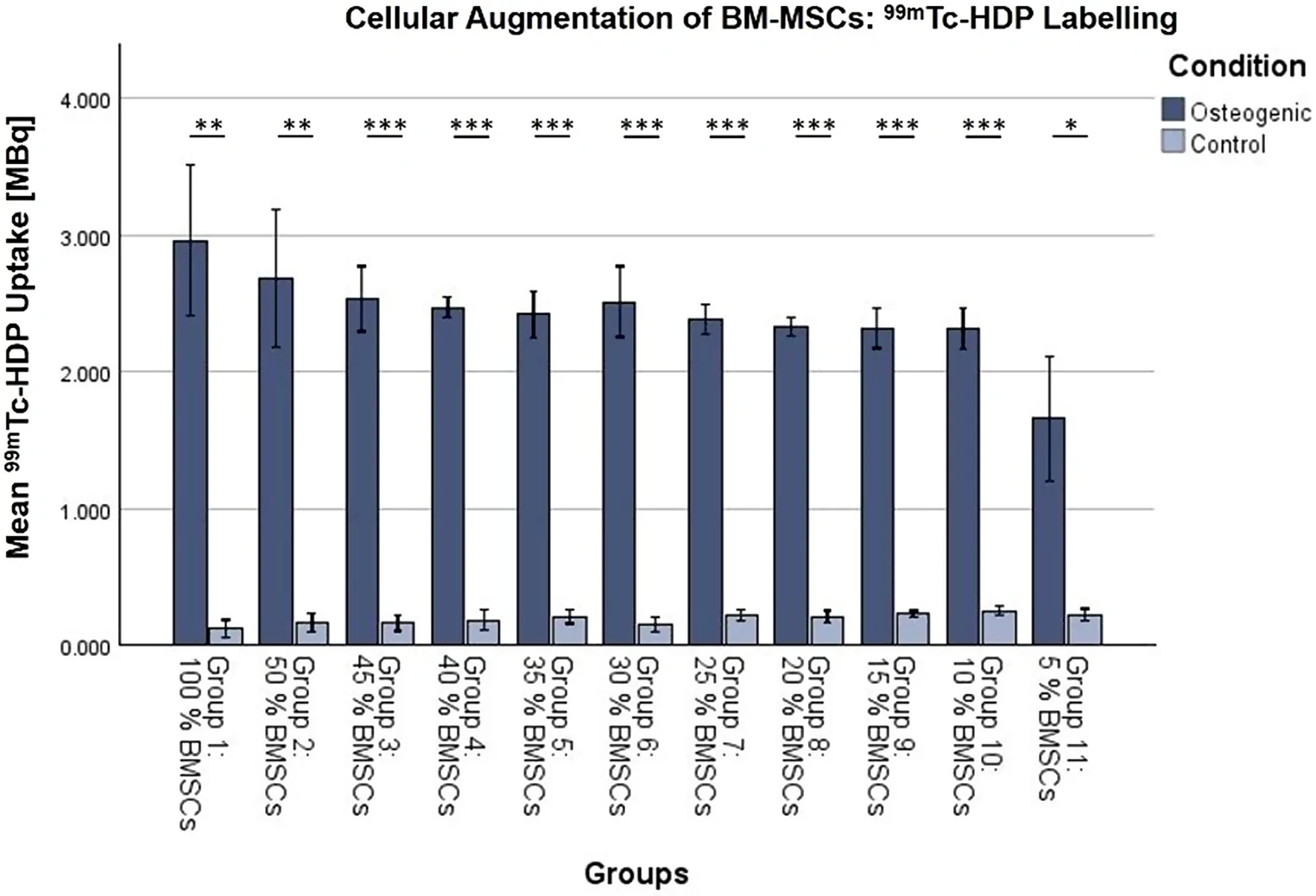
Results of the ^99m^Tc-HDP labelling for the assessment of the osteogenic potential of cellularly augmented BM-MSC cultures in vitro. The error bars represent the standard deviation. Stars over a horizontal line indicate a statistically significant between the connected groups. *: significance ≤ 0.05; **: significance *p* ≤ 0.01; ***: significance *p* ≤ 0.001

The ^99m^Tc-HDP uptake was generally higher in the osteogenic condition compared to the control condition for all 11 groups. Within the osteogenic condition, the uptake was highest for group 1 OM (B.100/A.0) with 2.957 MBq while it was lowest for group 11 OM (B.5/A.95) with 1.655 MBq. Between group 2 OM (B.50/A.50) and group 10 OM (B.10/A.90), the uptake was very similar with values between 2.681 MBq and 2.314 MBq. Within the control condition, the uptake was generally much lower while being highest for group 10 CTRL (B.10/A.90) with 0.252 MBq and lowest for group 1 CTRL (A.100/B.0) with 0.122 MBq (see Fig. 2).

The Welch ANOVA showed a p-value of *p* = 0.034 for the osteogenic condition and *p* = 0.044 for the control condition. Considering both p-values were larger than 0.025, there was no statistical significance within each condition which was confirmed by the Games-Howell post-hoc test. Therefore, no statistically significant difference between the osteogenic baseline group (group 1 OM (B.100/A.0)) and all other osteogenic groups (group 2 OM (B.50/A.50) – group 11 OM (B.5/A.95)) could be revealed. However, although no statistical significance could be detected, the data showed the trend of a slow decrease of the uptake in the osteogenic condition between group 1 OM (B.100/A.0; 2.957 MBq) and group 10 OM (B.10/A.90; 2.314 MBq) and then a more pronounced decrease towards group 11 OM (B.5/A.95; 1.655 MBq).

For each group, the osteogenic condition always showed a significantly higher uptake than the control condition (p-values between *p* = 0.04 and *p* < 0.001).

### Alkaline phosphatase activity

**Fig. 3:**
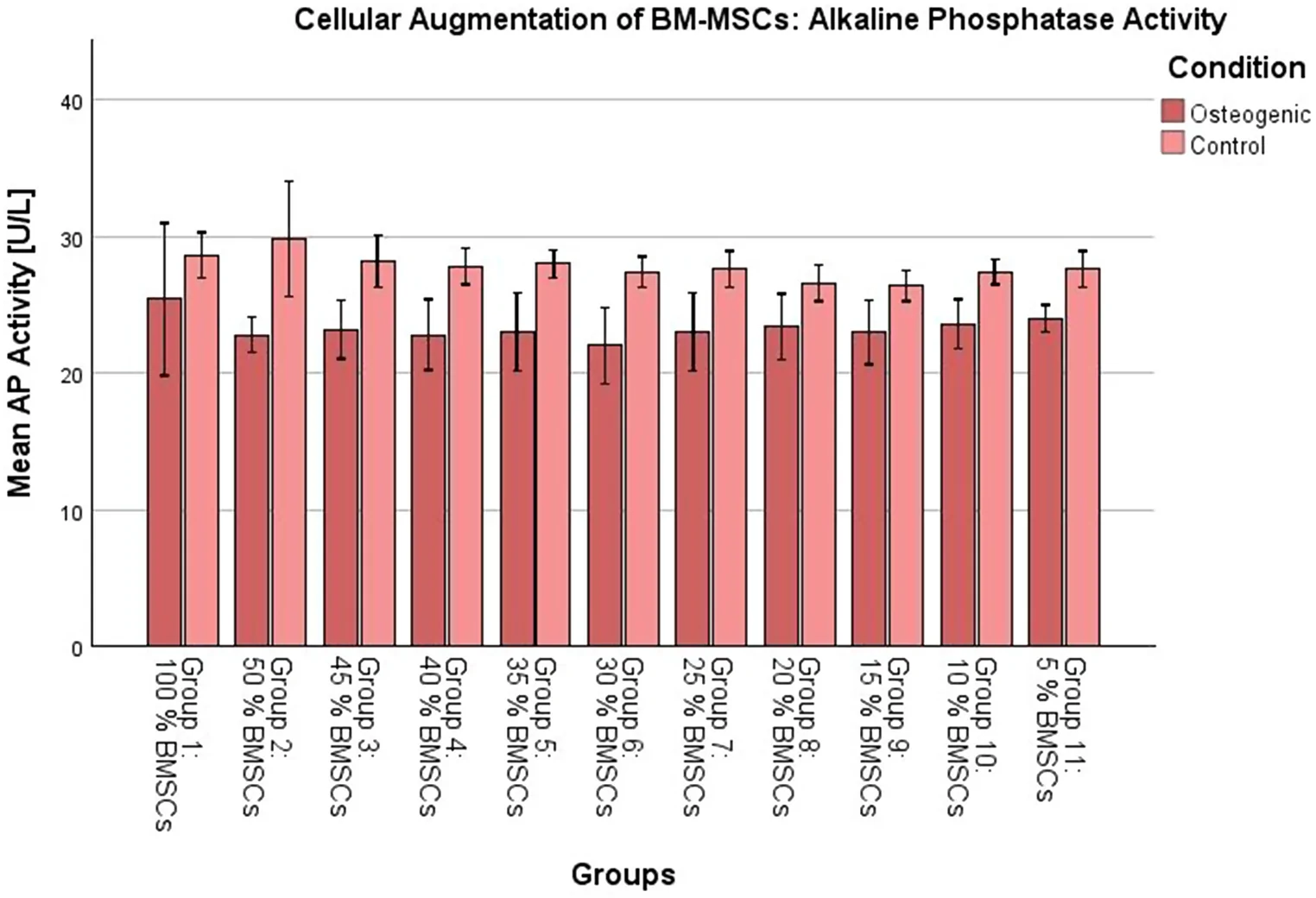
Results of the measurement of the alkaline phosphatase (AP) activity in the cell culture medium for the assessment of the osteogenic potential of cellularly augmented BM-MSC cultures in vitro. The error bars represent the standard deviation

Within both the osteogenic and the control condition, the values measured were very similar, ranging between 22.0 U/L (group 6 OM (B.30/A.70)) and 25.4 U/L (group 1 OM (B.100/A.0)) within the osteogenic condition and between 26.6 U/L (group 9 CTRL (B.15/A.85)) and 29.8 U/L (group 2 CTRL (B.50/A.50)) for the control condition (see Fig. 3).

The Welch-ANOVA identified no significant differences within both conditions (*p* = 0.927 for osteogenic and *p* = 0.507 for control condition). Consequently, no statistically significant difference between the osteogenic baseline group (group 1 OM (B.100/A.0)) and all other osteogenic groups (group 2 OM (B.50/A.50) – group 11 OM (B.5/A95)) could be revealed. For all groups, there was no significant difference between the alkaline phosphatase activity of the osteogenic condition compared to the control condition.

### Osteocalcin concentration

**Fig. 4:**
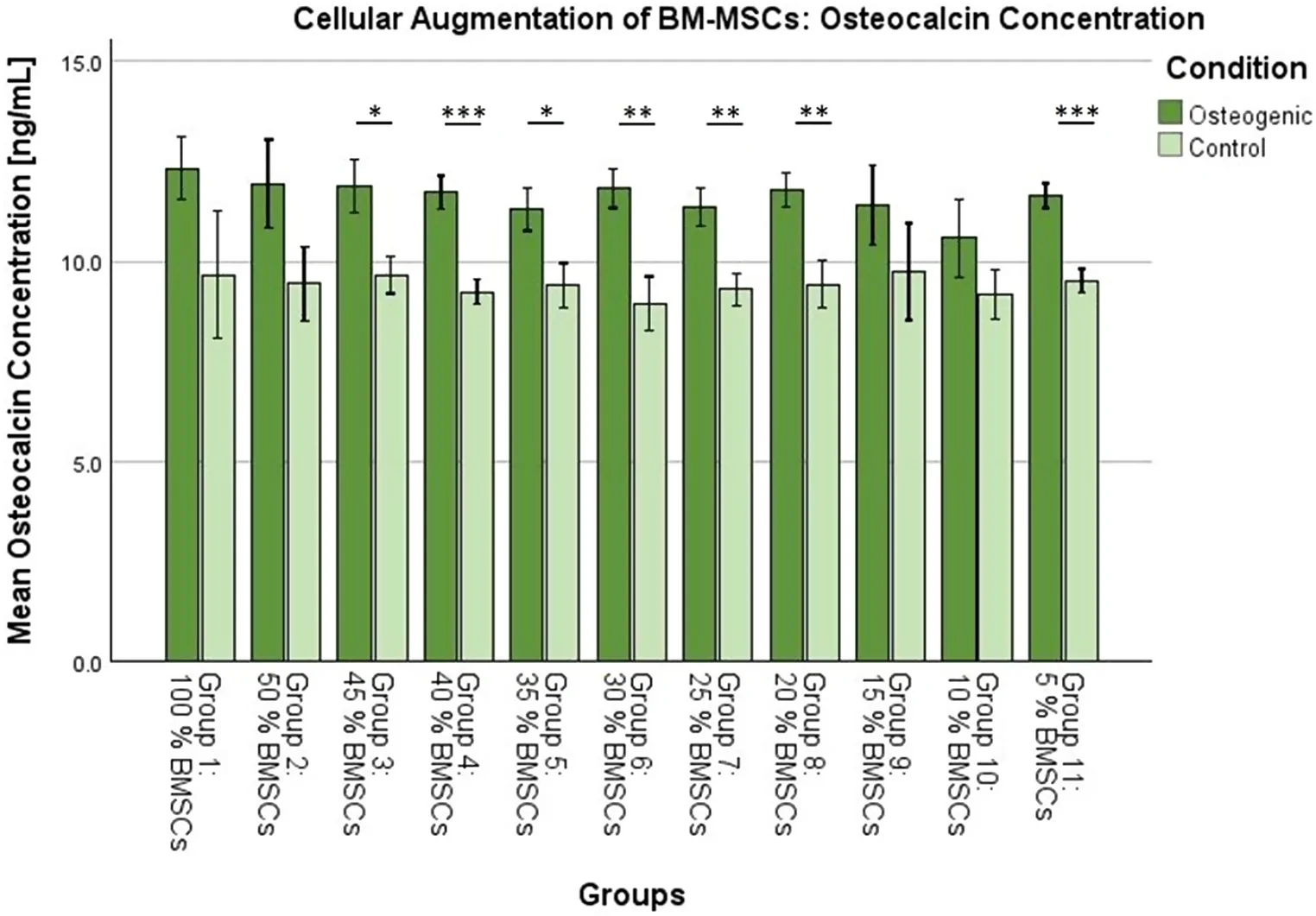
Results of the measurement of the osteocalcin concentration in the cell culture medium for the assessment of the osteogenic potential of cellularly augmented BM-MSC cultures in vitro. The error bars represent the standard deviation. Stars over a horizontal line indicate a statistically significant between the connected groups. *: significance ≤ 0.05; **: significance *p* ≤ 0.01; ***: significance *p* ≤ 0.001

The osteocalcin concentration was generally higher in the osteogenic condition than in the control condition. The measured values were between 10.6 ng/mL (group 10 OM (B.10/A.90)) and 12.3 ng/mL (group 1 OM (B100/A.0)) in the osteogenic condition and between 8.9 ng/mL (group 6 CTRL (B.30/A.70)) and 9.7 ng/mL (group 1 CTRL (B.100/A.0), group 3 CTRL (B.45/A.55) and group 9 CTRL (B.15/A.85)) in the control condition (see Fig. 4).

The conducted Welch-ANOVA analysis showed neither a statistically significant difference within the osteogenic condition (*p* = 0.358) nor in the control condition (*p* = 0.831). Therefore, no statistically significant difference between the osteogenic baseline group (group 1 OM (B.100/A.0)) and all other osteogenic groups (group 2 OM (B.50/A.50) – group 11 OM (B.5/A95)) could be revealed.

When testing for the differences between the osteogenic and control condition, we found that the osteocalcin concentration was significantly higher in the osteogenic condition compared to the control condition for all groups with the exceptions of group 1 (B.100/A.9; *p* = 0.330), group 2 (B.50/A.50; *p* = 0.164), group 9 (B.15/A.85; *p* = 0.706) and group 10 (B.10/A.90; *p* = 0.548).

### Calcium concentration

**Fig. 5:**
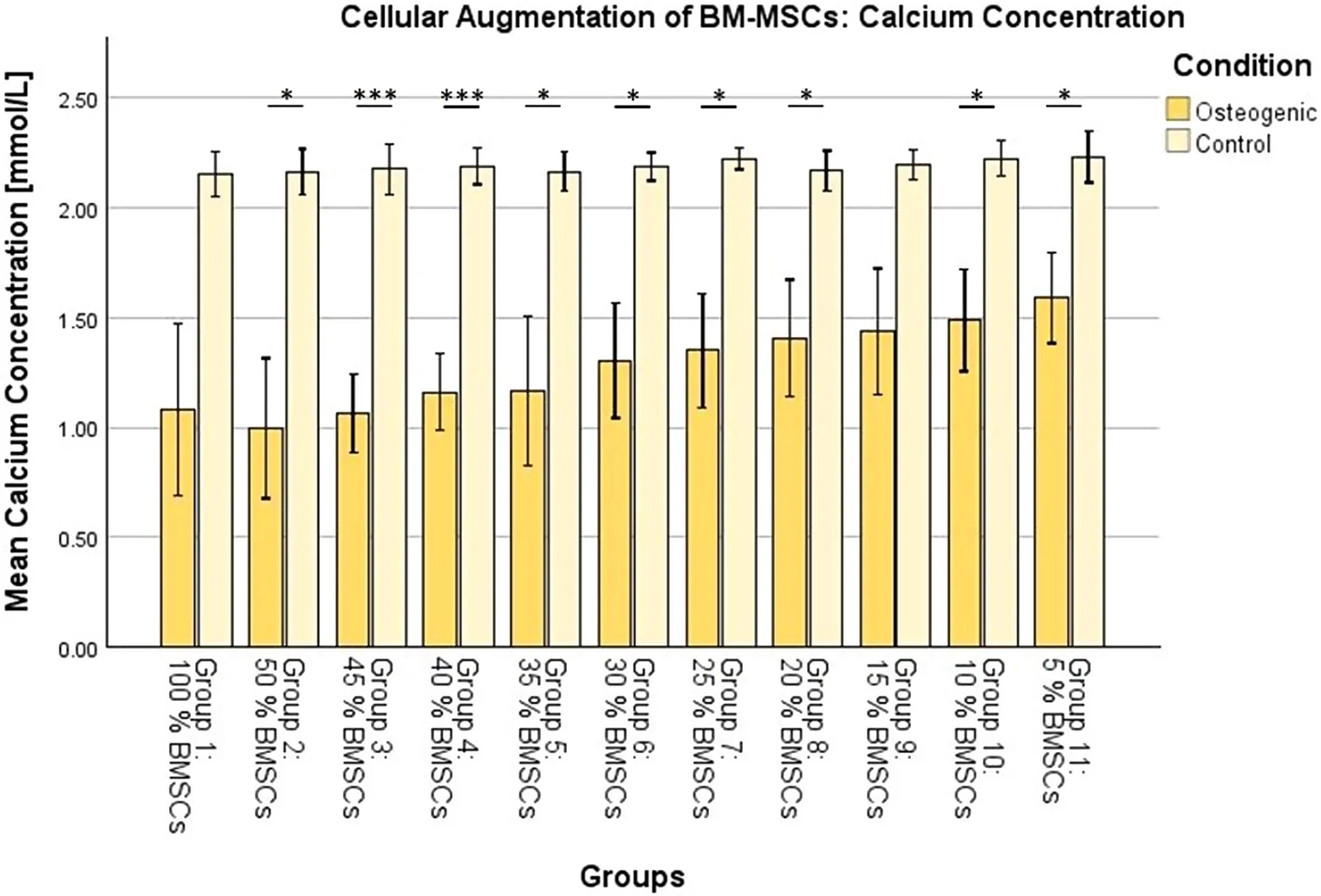
Results of the measurement of the calcium concentration in the cell culture medium for the assessment of the osteogenic potential of cellularly augmented BM-MSC cultures in vitro. The error bars represent the standard deviation. Stars over a horizontal line indicate a statistically significant between the connected groups. *: significance ≤ 0.05; ***: significance *p* ≤ 0.001

The calcium concentration measured in the cell culture medium of the osteogenic condition after 3 weeks was generally lower than the concentration measured in the control condition. The values for the osteogenic condition ranged between 1.00 mmol/L (group 2 OM (B.50/A.50)) and 1.59 mmol/L (group 11 OM (B.5/A.95)), while the values for the control condition were between 2.15 mmol/L (group 1 CTRL (B.100/A.0)) and 2.23 mmol/L (group 11 CTRL (B.5/A.95)) (see Fig. 5).

When comparing the osteogenic condition to the control condition for each group, we found statistically significant differences between the two conditions for all groups with the exception of group 1 (B.100/A.0; *p* = 0.059) and group 9 (B.15/A.85; *p* = 0.068).

Generally, the osteogenic condition showed a slow increase of the calcium concentration between group 1 OM (B.100/A.0; 1.08 mmol/L) and group 11 OM (B.5/A.95; 1.59 mmol/L), although no statistically significant difference between the osteogenic baseline group (group 1 OM (B.100/A.0)) and all other osteogenic groups (group 2 OM (B.50/A.50) – group 11 OM (B.5/A95)) could be revealed in the Welch-ANOVA (*p* = 0.045). The Welch-ANOVA also showed no differences within the control condition (*p* = 0.932).

### Phosphate concentration

**Fig. 6:**
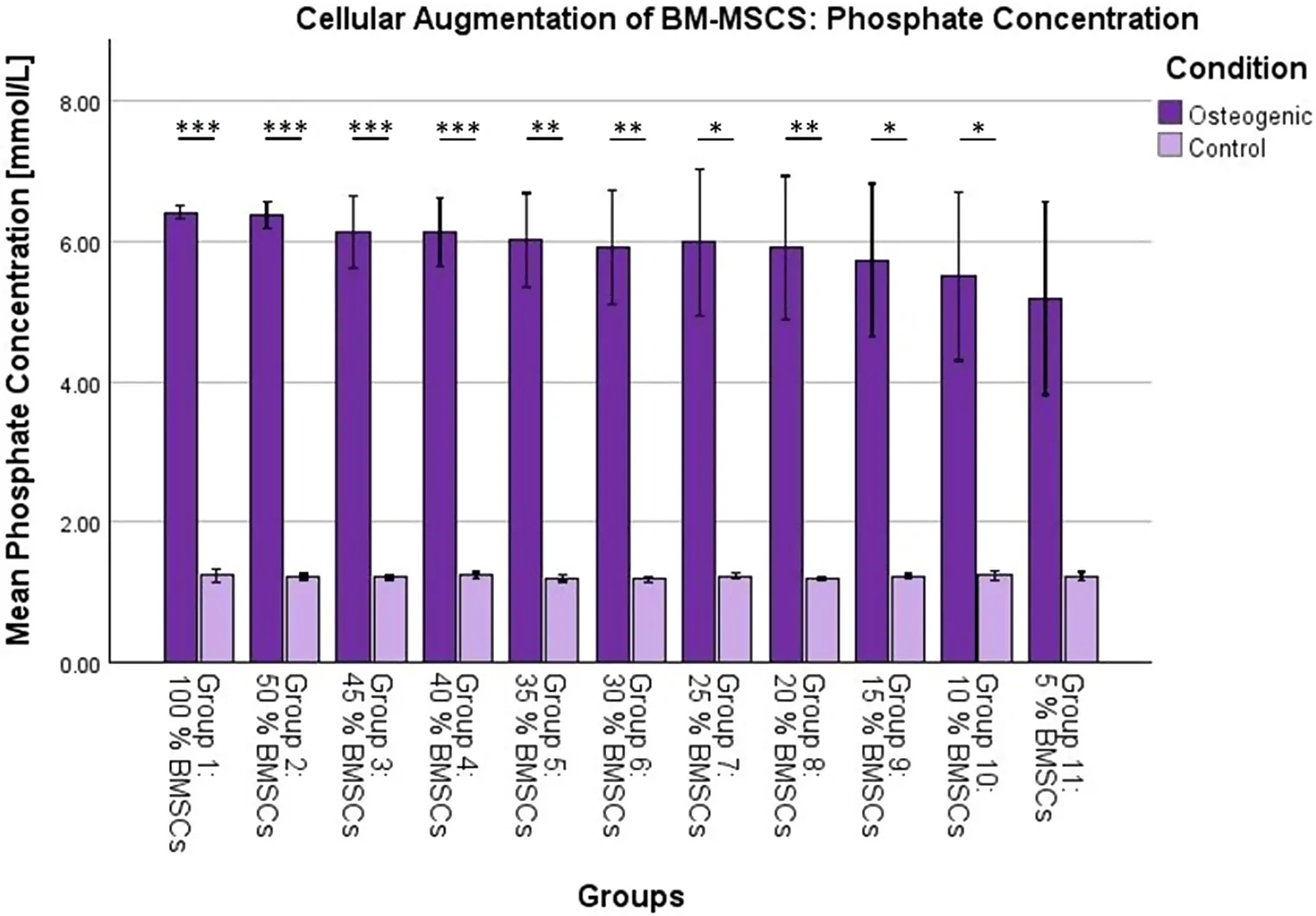
Results of the measurement of the phosphate concentration in the cell culture medium for the assessment of the osteogenic potential of cellularly augmented BM-MSC cultures in vitro. The error bars represent the standard deviation. Stars over a horizontal line indicate a statistically significant between the connected groups. *: significance ≤ 0.05; **: significance *p* ≤ 0.01; ***: significance *p* ≤ 0.001

The phosphate concentration measured in the cell culture medium of the osteogenic condition after 3 weeks was generally higher than the concentration measured in the control condition. The phosphate concentration in the osteogenic condition was lowest with 5.20 mmol/L for group 11 OM (B.5/A.95) and highest with 6.42 mmol/L for group 1 OM (B.100/A.0) and showed a steady increase between groups 1 OM and 11 OM. Within the control condition, the values measured were very similar between the groups and ranged from 1.19 mmol/L (group 6 CTRL (B.30/A.70)) and 1.25 mmol/L (group 4 CTRL (B.40/A.60)) (see Fig. 6).

In the Welch-ANOVA, no statistically significant difference between the osteogenic baseline group (group 1 OM (B.100/A.0)) and all other osteogenic groups (group 2 OM (B.50/A.50) – group 11 OM (B.5/A95)) could be revealed (*p* = 0.367). There was also no significant difference between any groups of the control condition (*p* = 0.718). Regarding visible trends, groups 1 OM (B.100/A.0; 6.42 mmol/l) to 7 OM (B.25/A.75; 5.99 mmol/L) showed similar phosphate concentrations, while there was then a slow decline towards group 11 (B.5/A.95; 5.20 mmol/L).

The comparison of the osteogenic and control conditions demonstrated significantly higher phosphate concentrations in the osteogenic condition compared to the control condition for all groups except for group 11 (B.5/A.95; *p* = 0.057).

### Summary: non-destructive assessment of functional osteogenic potential

The labelling with the radioactive tracer ^99m^Tc-HDP, the measurement of alkaline phosphatase activity as well as the measurement of osteocalcin, calcium und phosphate concentrations in the cell culture supernatant can all be considered non-destructive assessment methods of the functional osteogenic potential, as they do not require a destruction of the 2D cell layer. In none of these assessments, a statistically significant difference between the osteogenic baseline culture (group 1 (B.100/A.0)) and any of the augmented cultures (group 2 (B.50/A.50) – group 11 (B.5/A.95)) could be detected.

The age of the donors did not have a significant impact on the non-destructive measurements of the osteogenic potential in both the osteogenic and control condition with two exceptions: the measurement of osteocalcin in the osteogenic condition (*p* = 0.029) and the measurement of phosphate in the osteogenic condition (*p* = 0.023) (see Table 4). The gender of the donors did not have any influence on the measurements (see Table 5).

**Table 4:** Influence of age on the osteogenic potential

Non-destructive measurement of osteogenic potential	Result of the linear mixed model for age	
	Osteogenic Condition	Control Condition
^99m^Tc-HDP uptake	*p* = 0.232	*p* = 0.076
Alkaline phosphatase activity	*p* = 0.634	*p* = 0.208
Osteocalcin concentration	*p* = 0.029	*p* = 0.287
Calcium Concentration	*p* = 0.155	*p* = 0.909
Phosphate Concentration	*p* = 0.023	*p* = 0.706

**Table 5:** Influence of gender on the osteogenic potential

Non-destructive measurement of osteogenic potential	Result of the linear mixed model for gender	
	Osteogenic Condition	Control Condition
^99m^Tc-HDP uptake	*p* = 0.296	*p* = 0.731
Alkaline phosphatase activity	*p* = 0.903	*p* = 0.251
Osteocalcin concentration	*p* = 0.432	*p* = 0.063
Calcium Concentration	*p* = 0.838	*p* = 0.918
Phosphate Concentration	*p* = 0.527	*p* = 0.854

### Alizarin Red staining

**Fig. 7:**
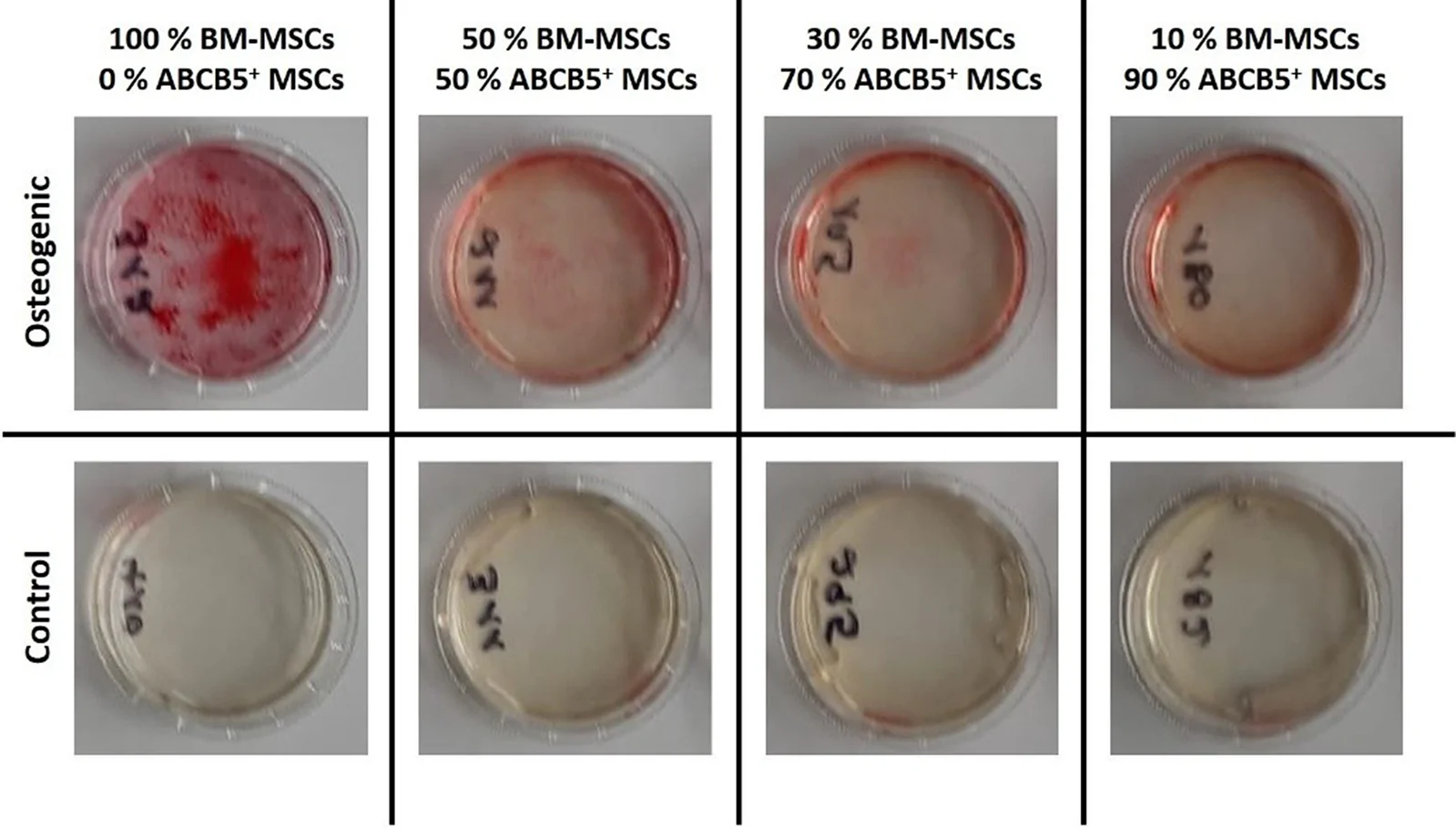
Macroscopic images of the cell culture dishes after staining with alizarin red

**Fig. 8:**
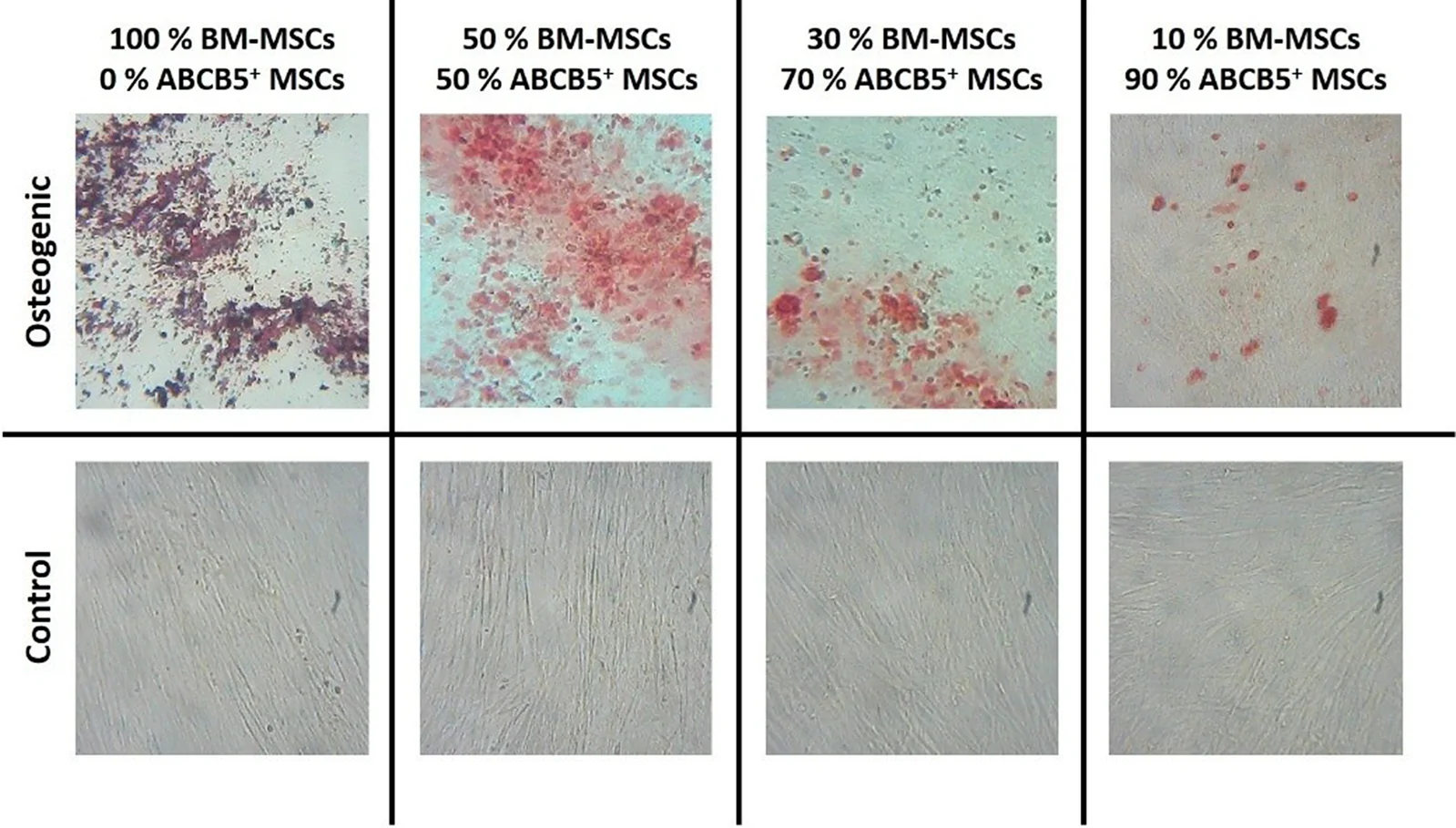
Exemplary light microscopy images of the center of the dishes after staining with Alizarin Red (50x magnification)

The qualitative Alizarin Red staining demonstrated no staining in the control condition of groups 1 CTRL (B.100/A.0), 2 CTRL (B.50/A.50), 6 CTRL (B.30/A.70) and 10 CTRL (B.10/A.90). Regarding the osteogenic condition, two main trends were observable (see Fig. 7): Between group 10 OM (B.10/A.90) and group 1 OM (B.100/A.0) the intensity of the staining increased with the greatest increase between group 2 OM (B.50/A.50) and group 1 OM (B.100/A.0). At the same time, the organization of the mineral grew stronger towards group 1 OM (B.100/A.0) with an increasingly organized mineralized area in the center of the dishes. Again, the greatest difference could be observed between group 2 OM (B.50/A.50) and group 1 OM (B.100/A.0). When looking at the center of each dish under a light microscope, both macroscopically describable trends could be confirmed (see Fig. 8).

The photometric quantification confirmed the highest intensity of the Alizarin Red staining for group 1 OM (B.100/A.0) compared to all other groups. However, at the same time it also did not find any difference between group 2 OM (B.50/A.50), group 6 OM (B.30/A.70) and group 10 OM (B.10/A.90) regarding extinction. The control condition of every group measured showed only very little extinction (see Table 6).

**Table 6:** Alizarin Red staining, quantification - extinction at 405 nm

Group	Condition	Extinction at 405 nm
Group 1 (B.100/A.0)	Osteogenic (OM)	3.178
	Control (CTRL)	0.216
Group 2 (B.50/A.50)	Osteogenic (OM)	1.365
	Control (CTRL)	0.435
Group 6 (B.30/A.70)	Osteogenic (OM)	1.188
	Control (CTRL)	0.409
Group 10 (B.10/A.90)	Osteogenic (OM)	1.460
	Control (CTRL)	0.410

### Energy dispersive X-Ray spectroscopy (EDX)

The calcium to phosphate ratio showed the following results for the specimen measured (see Table 7):

**Table 7:** Results of the EDX-analysis. As an example, the SEM-image of the calcium deposition in group 2 OM (B.50/A.50) has been included on the right

Group	Calcium/Phosphate Ratio	
1 OM (B.100/A.0)	1.2	
2 OM (B.50/A.50)	1.1	
10 OM (B.10/A.90)	0.3	

## Discussion

### Definition of the functional osteogenic potential

The term “osteogenic potential” is not well defined in literature, but it can be considered cells’ ability to form and mineralize a bone-specific extracellular matrix. As the formation of mature mineral is both the end stage of osteogenesis as well as the deciding factor for the mechanical strength of the bone tissue, a “functional osteogenic potential” can be defined based on the amount of calcium phosphate mineral formed [29]. A number of markers exist to monitor and confirm this process in vitro, including the following:

Mesenchymal stem cells undergoing osteogenic differentiation express alkaline phosphatase (AP) at an early stage [30]. This enzyme catalyzes the hydrolyzation of larger phosphate molecules, such as β-glycerol phosphate, leading to the liberation of free phosphate (P_i_) [31]. Both the enzymatic activity of AP as well as the amount of free phosphate in the cell culture media are therefore markers of the osteogenic activity in vitro.

P_i_ in the cell culture medium is rinsed from the culture at every medium change unless it has formed insoluble compounds. In osteogenic cultures, P_i_ mainly forms complexes with calcium ions, leading to the aggregation of different calcium-phosphate molecules. This happens as a natural chemical reaction at first, resulting in amorphous calcium-phosphates with a high percentage of phosphate [32]. The process is later amplified and controlled by the cells undergoing osteogenic differentiation, leading to the formation of specific calcium-complexes, most dominantly hydroxyapatite [33]. The reaction of P_i_ with free calcium from the cell culture medium results in a reduction of calcium concentration in the medium during this process. Therefore, the measurement of free calcium can be used as an osteogenic marker in vitro as well [34].

Furthermore, the insoluble calcium-phosphate molecules can be assessed directly. This can happen either by radioactive ^99m^Tc-polyphosphate labelling or alizarin red staining. While both methods measure crystalline calcium-phosphate, their principle of detection differs. The polyphosphates bound to ^99m^Tc bind to phosphate groups by means of adsorption, meaning that this assessment is able to detect early insoluble mineral with a high phosphate but low calcium percentage [35]. Meanwhile, alizarin red forms complexes with calcium, therefore detecting later stages of mineral formation [36]. While both measurement methods correlate highly with each other for maturer cell cultures, they can differ, especially during early osteogenesis, when ^99m^Tc-HDP labelling detects significant differences between osteogenic and control cultures much earlier than alizarin red staining [25, 26]. Both methods are not specific for hydroxyapatite. They can therefore be combined them with an EDX-analysis which can detect the ratio of calcium and phosphate in the mineral. While pure hydroxyapatite would demonstrate a calcium to phosphate ratio of 1.67, the calcium-phosphate found in human bone has a larger range, showing calcium to phosphate ratios between 1.39 and 1.62 [37].

Osteocalcin as a protein of the extracellular matrix is not part of the mineral formed. However, since it serves to align the mineral and bind it to the collagen of the matrix, the amount of free osteocalcin in the cell culture medium has also been assessed for the purpose of this study [38].

### ^99m^Tc-HDP labelling, phosphate concentration and alkaline phosphatase activity

Both the ^99m^Tc-HDP uptake and the phosphate concentration in the cell culture medium were, at least within the osteogenic condition, highest for group 1 OM (100% BM-MSCs/0% ABCB5^+^ MSCs), before slowly decreasing as ABCB5^+^ MSC augmentation percentage went up, finally reaching their lowest value for group 11 OM (5% BM-MSCs/95% ABCB5^+^ MSCs). While this trend did not reach statistical significance in the Welch-ANOVA, it should still be considered bearing in mind that the study’s sample size was comparably small with *n* = 5. It appears, that the P_i_ liberation (measured as free phosphate in the medium) and subsequent precipitation (measured by labelling with ^99m^Tc-HDP) are at least to a degree dependent on the BM-MSCs content of a cell culture. One possible explanation for this phenomenon could be a higher AP activity of BM-MSCs. However, when looking at the AP activity measured in the cell culture medium supernatant under the osteogenic condition, neither a statistically significant difference between any groups nor a relevant trend could be detected. This might be due to the fact, that AP is mainly a membrane-bound protein and is only secreted into the medium in small quantities [30]. These small quantities are not even great enough to achieve a higher AP activity in the medium of the osteogenic condition compared to the control condition. Instead, the AP activity in the control condition is generally slightly higher, which can be explained by the intrinsic AP activity of the FSC which was added to the cell culture medium [30]. While this extrinsic AP activity was present in both the osteogenic as well as the control condition it probably had a stronger influence on the AP levels in the medium of the osteogenic condition. This is because the presence of AP in a cellular environment suppresses these cells intrinsic synthesis of AP to a higher degree when the cells are undergoing osteogenic differentiation [30]. As free AP has a half-life of only a few hours, the analysis of the cell culture medium two days after the last medium change can only detect the recently secreted AP, which would then be lower for the osteogenic condition [39]. Overall, using AP activity in vitro to assess functional osteogenic potential has some serious limitations, prohibiting it to allow definitive conclusions regarding the functional osteogenic potential in the context of this study.

On the other hand, only little difference can be shown between the osteogenic baseline culture group 1 OM (100% BM-MSCs/0% ABCB5^+^ MSCs) and the first augmented osteogenic culture group 2 OM (50% BM-MSCs/50% ABCB5^+^ MSCs) regarding ^99m^Tc-HDP uptake (2.96 MBq vs. 2.68 MBq) and phosphate concentration (6.42 mmol/L vs. 6.38 mmol/L), indicating that 50% of BM-MSCs can be replaced by ABCB5^+^ MSCs without compromising phosphate liberation and precipitation.

### Alizarin Red staining, calcium concentration and EDX

The staining with Alizarin Red is generally considered the gold standard for the assessment of the osteogenic potential in vitro and is even part of the definition of mesenchymal stem cells as stated by the International Society for Cellular Therapy [15]. In this study, the Alizarin Red staining revealed a higher amount of calcium phosphate mineral in the osteogenic condition compared to the control condition in the representative dishes measured. At the same time, it showed that the amount of calcium-based mineral synthesized in the osteogenic baseline culture group 1 OM (100% BM-MSCs/ 0% ABCB5^+^ MSCs) was much higher compared to the osteogenic augmented cultures group 2 (50% BM-MSCs/50% ABCB5^+^ MSCs), group 6 (30% BM-MSCs/70% ABCB5^+^ MSCs) and group 10 (10% BM-MSCs/90% ABCB5^+^ MSCs). The osteogenic augmented cultures themselves were relatively similar to each other. The calcium concentration in the medium confirms the trend of a stronger precipitation of calcium for groups with a lower percentage of ABCB5^+^ MSCs within the osteogenic condition but it does not identify such a stark contrast between group 1 OM (100% BM-MSCs/ 0% ABCB5^+^ MSCs) and group 2 OM (50% BM-MSCs/50% ABCB5^+^ MSCs), whose calcium concentration values differ only marginally and are in fact lower for group 2 OM than for group 1 OM (1.08 mmol/L vs. 1.00 mmol/L). Of course, the calcium concentration in the medium is only a snapshot of the whole process and only describes the situation between the end of the cell culture and the last medium change two days prior. Still, in the EDX analysis the mineral composition is also almost identical between group 1 OM (100% BM-MSCs/ 0% ABCB5^+^ MSCs) and group 2 OM (50% BM-MSCs/50% ABCB5^+^ MSCs) with calcium to phosphate ratios of 1.2 and 1.1, respectively.

Overall, it seems that while the quantity of mineralization is highest for group 1 OM (100% BM-MSCs/ 0% ABCB5^+^ MSCs) by some margin, the quality of the mineralization represented by the matrix composition places group 2 OM (50% BM-MSCs/50% ABCB5^+^ MSCs) closer to the osteogenic baseline culture than the other end of the spectrum of augmented cultures, specifically group 10 OM (10% BM-MSCs/90% ABCB5^+^ MSCs) which showed a calcium to phosphate ratio of just 0.3 in the EDX analysis.

### Osteocalcin concentration

Besides the formation of inorganic mineral, osteogenesis also includes the formation of an organic extracellular matrix [40]. This matrix consists of typical proteins, such as osteocalcin, which can be measured in the cell culture medium and serve as a marker for later osteogenesis in-vitro [41]. However, most osteocalcin is bound to the extracellular matrix where it, among other things, is responsible to aligning the inorganic mineral along the collagen fibers, thus structuring the entire matrix [38]. Similar to AP, only a small amount of osteocalcin is secreted into the matrix, where it can be measured non-destructively. While this allows to identify successful osteogenic differentiation, shown by the generally higher osteocalcin concentration in the osteogenic condition of all groups in this study, the sensitivity is relatively low, which is demonstrated by the fact that for 4 of 11 groups the difference between osteogenic and control condition did not reach statistical significance. Generally, the sensitivity of secretory osteocalcin as an osteogenic marker does not seem sensitive enough to identify trends regarding the osteogenic potential of augmented cell cultures.

### Influence of age and gender on the osteogenic potential

To identify any relevant influence of age or gender on the non-destructive measurements of the osteogenic potential, a linear mixed model was performed. Regarding the influence of gender, no significances could be detected for any group. Similarly, the donors’ age did not have a significant influence on most groups. Two exceptions were posed by osteocalcin in the osteogenic condition, where osteocalcin concentration correlated positively with age (*r* = 0.881, *p* = 0.048), as well as phosphate in the osteogenic condition. The fact that osteocalcin concentration in the cell culture medium correlated positively with age, meaning older donors showed a higher mean osteocalcin concentration in the cell culture medium, is consistent with the available literature, which shows a slight increase of osteocalcin concentration with higher age [42]. Literature also demonstrates an influence of age on the serum phosphate level, although the effect is more complex in nature, especially for female patients [43]. Consequently, the correlation between age and phosphate concentration in the cell culture medium did not reach significance in this study (*r* = -0.579, *p* = 0.306).

While the donors age did have a statistical influence on the osteocalcin and phosphate concentration in the cell culture medium for the osteogenic condition, it is unlikely that this influence invalidated the findings, as the same donors were used for all groups, meaning that the age influence impacted all groups. Still, it can be assumed that the osteogenic potential is, to an extent, a function of patient age.

### Outlook

This study provides proof-of-concept that augmentation/substitution of BM-MSCs with allogeneic ABCB5^+^ MSCs is feasible in an in-vitro setting without significant reduction of osteogenic potential compared to pure BM-MSC cultures. It is now the task of future research to generate further insight into the crosstalk of BM-MSCs and ABCB5^+^ MSCs during osteogenesis.

MSCs are strong modulators of the human immune response [44]. ABCB5^+^ MSCs, for example, can induce a macrophage switch from pro-inflammatory M1 to anti-inflammatory M2 macrophages [20]. This immune activation will become especially relevant in future in-vivo studies, where BM-MSCs and ABCB5^+^ MSCs are interacting not only with one another, but with entirely different populations of cells as well. However, it can be assumed that the anti-inflammatory properties of ABCB5^+^ MSCs can shorten the initial inflammatory phase of osteoregeneration and therefore accelerate bone formation [45].

## Summary and clinical relevance

Among clinicians it is well known and accepted that the osteogenic potential of bone grafts is dependent on the concentration of biologically active cells within these grafts [9]. For the clinical practice, it is important to balance the need for a sufficient concentration of cells with a sufficient volume of the graft. Especially in the case of re-grafting, the availability of material and therefore the volume of the graft can be limited. For example, it is known that a re-harvest of the posterior iliac crest becomes possible in humans not until 2 years after the initial procedure [46]. Therefore, to increase the demanded overall volume, an augmentation of the graft with established acellular graft materials such as tricalcium phosphate is possible and common [47]. However, this leads to a dilution of the cellular fraction, decreasing osteogenic potential and practically setting an upper limit for acellular graft augmentation at 30% overall volume [2]. A promising alternative could be the utilization of allogenic mesenchymal stem cells which would be almost limitlessly available and which do contain a high regenerative potential [10].

In this study, we assessed the possibility of augmenting BM-MSC cell cultures with allogenic ABCB5^+^ MSCs by measuring the functional osteogenic potential in different ways. In summary, we found that the augmentation has an impact on all aspects of the osteogenic potential we looked at (^99m^Tc-HDP uptake, phosphate, calcium and osteocalcin concentration in cell culture medium, alkaline phosphatase activity in cell culture medium, Alizarin Red staining, mineral composition in EDX) depending on the cell fractions. While no statistically significant difference could be detected between any groups within the osteogenic condition when looking at the non-destructive measurements (i.e. ^99m^Tc-HDP uptake, phosphate, calcium and osteocalcin concentration in cell culture medium, alkaline phosphatase activity in cell culture medium), the observable trends suggest a slight decrease in functional osteogenic potential as the level of augmentation rises, indicating a certain amount of dilution of the osteogenic potential even in case of a cellular augmentation. However, between the osteogenic baseline culture, which was not augmented at all, and the culture which was augmented with 50% ABCB5^+^ MSCs, there was no relevant difference regarding the values measured. This also includes the mineral composition as observed in the EDX. While the absolute quantity of mineral is still highest in the baseline osteogenic culture, as shown by the Alizarin Red staining, the functional osteogenic potential between the baseline osteogenic culture and the osteogenic culture augmented with 50% ABCB5^+^ MSC appears very similar.

We therefore concluded that an augmentation of 2D BM-MSC cultures with ABCB5^+^ MSCs in vitro is possible up to 50% of the overall cell content. This value is already higher than the 30% augmentation achievable with acellular materials and could perhaps even grow further under optimized conditions in 3D. Further studies with larger sample sizes and ultimately animal models are required to confirm these findings. Still, we believe that this study can serve as the basis for the development of biologically augmented bone grafts into a successful and reliable treatment option for bone non-unions and bone defects.

## Data Availability

All data that support the findings of this study are available from the corresponding author upon reasonable request.
